# Metabolic Profiling of Systemic Lupus Erythematosus and Comparison with Primary Sjögren’s Syndrome and Systemic Sclerosis

**DOI:** 10.1371/journal.pone.0159384

**Published:** 2016-07-21

**Authors:** Anders A. Bengtsson, Johan Trygg, Dirk M. Wuttge, Gunnar Sturfelt, Elke Theander, Magdalena Donten, Thomas Moritz, Carl-Johan Sennbro, Frida Torell, Christian Lood, Izabella Surowiec, Stefan Rännar, Torbjörn Lundstedt

**Affiliations:** 1 Department of Rheumatology, University Hospital Lund, Lund, Sweden; 2 Computational Life Science Center (CLiC), Department of Chemistry, Umeå University, Umeå, Sweden; 3 Department of Rheumatology, Skåne University Hospital Malmö, Lund University, Lund, Sweden; 4 AcureOmics AB. Tvistevägen 48, Umeå, Sweden; 5 Swedish Metabolomics Centre, Swedish University of Agricultural Sciences, Umeå, Sweden; 6 Leo Pharma A/S, 55 Industriparken, DK-2750 Ballerup, Denmark; Korea University, REPUBLIC OF KOREA

## Abstract

Systemic lupus erythematosus (SLE) is a chronic inflammatory autoimmune disease which can affect most organ systems including skin, joints and the kidney. Clinically, SLE is a heterogeneous disease and shares features of several other rheumatic diseases, in particular primary Sjögrens syndrome (pSS) and systemic sclerosis (SSc), why it is difficult to diagnose The pathogenesis of SLE is not completely understood, partly due to the heterogeneity of the disease. This study demonstrates that metabolomics can be used as a tool for improved diagnosis of SLE compared to other similar autoimmune diseases. We observed differences in metabolic profiles with a classification specificity above 67% in the comparison of SLE with pSS, SSc and a matched group of healthy individuals. Selected metabolites were also significantly different between studied diseases. Biochemical pathway analysis was conducted to gain understanding of underlying pathways involved in the SLE pathogenesis. We found an increased oxidative activity in SLE, supported by increased xanthine oxidase activity and an increased turnover in the urea cycle. The most discriminatory metabolite observed was tryptophan, with decreased levels in SLE patients compared to control groups. Changes of tryptophan levels were related to changes in the activity of the aromatic amino acid decarboxylase (AADC) and/or to activation of the kynurenine pathway.

## Introduction

Systemic lupus erythematosus (SLE) is a chronic inflammatory systemic autoimmune disease affecting mostly females in childbearing ages. Most organ systems can be involved and symptoms from skin and joints are common but more severe disease with inflammation in kidneys and/or central nervous system (CNS) also occur frequently, and there is an increased mortality in this disease. The prevalence in Sweden has been estimated to 68/100,000 and the annual incidence to approximately 5/100,000 [1].

The complex pathogenesis of SLE is characterized by immunological abnormalities including involvement of T-cells, dendritic cells and B-cell hyperactivity with auto-antibody production resulting in the formation of immune complexes causing organ damage in host tissues, but there is still a lack of knowledge of what immune-pathological pathways are involved [2]. Consequently, the biomarkers used today to diagnose and to monitor disease activity in this heterogeneous disease are far from perfect with respect to sensitivity and specificity [3]. The SLE diagnosis is based on typical clinical manifestations with signs and symptoms from more than one organ system in combination with presence of autoantibodies [4]. The time course of the disease is characterized by alternating periods of flares and remissions. SLE is a chronic inflammatory disease where early treatment can prevent irreversible organ damage, poor quality of life and even mortality. Finding reliable biomarkers for early diagnosis of the disease and for the monitoring of disease activity would greatly facilitate the healthcare of SLE patients and optimization of treatment. This lack of markers has also hampered the efforts to monitor and evaluate the effects of novel therapeutics in clinical trials.

Diagnosing SLE can be a challenge even for experienced rheumatologists since symptoms are sometimes subtle and can also overlap with similar systemic autoimmune diseases, especially primary Sjögren´s syndrome (pSS) or systemic sclerosis (SSc) and might also be linked to rheumatoid arthritis. Furthermore, patients can be stratified into clinical phenotypic subsets, reflecting disease severity, which sometimes changes over time [5]. Considering the complexity of SLE, it is reasonable to argue that more than one biomarker will be required in order to reflect all aspects of SLE. Biomarker profiles that mirror clinical subsets of SLE would contribute to increased understanding of pathogenesis and perhaps also to more individualized treatment regimens.

Oxidative stress is one of the key factors in development and progression of SLE involved in several important pathways including immune system regulation and modification of autoantigens [6]. Neutrophils, a major contributor of reactive oxygen species (ROS), have consistently been reported to be hyper activated in SLE patients [7], and release proteolytic enzymes and reactive intermediates to induce tissue inflammation [8–10]. Furthermore, neutrophil-derived ROS, or ROS-inducing enzymes have been described to modify DNA enabling it to resist nuclease digestion and induce inflammation through the cytosolic cGAS-STING pathway [11] as well as oxidizing lipids promoting atherosclerosis [12]. Although more and more is known about pathogenetic pathways in SLE such as those involved in oxidative stress, this knowledge has not yet been transformed into useful biomarkers.

Metabolic profiling (metabolomics) is one approach that could be of help in the diagnosis of SLE but could possibly also be of use in monitoring of disease activity and support more individualized treatment regimens which will be increasingly important with new treatments available. The concept of metabolomics include the detection and quantification of low molecular weight molecules (metabolites) e.g. in a human subject. Metabolic profiling has been used to identify biomarkers for several diseases [13–19]. The fundamental rationale in metabolomics is that perturbations in a biological system, for instance, those caused by a disease, will be detectable as changes in concentrations of certain metabolites. In some cases (for example, congenital metabolic diseases), it may be possible to identify a single, robust diagnostic metabolite, but in many cases the perturbations are more subtle, involving the activation of multiple enzymatic (biochemical) pathways. In such cases, it is unlikely that any single biomarker will be sufficiently specific for diagnostic purposes. However, by using multivariate statistics, it may be possible to describe changes in patterns of biomarkers, rather than in individual biomarkers, that are highly discriminatory for the perturbation and/or disease state [19]. An additional advantage of metabolomics strategy is the possibility of revealing underlying biochemical phenomena associated with the disease, thus providing insights that help the development of a better understanding of the disease pathogenesis.

The aim of the present study was to conduct metabolic profiling on serum samples from SLE patients and discriminate them against controls as well as patients with closely related diagnoses namely pSS and SSc. Biochemical pathway analysis of metabolites differentiating SLE patients from controls was conducted aiming to gain better understanding of disturbed biochemical pathways in SLE disease.

## Materials and Methods

### Patients

Thirty SLE patients, all fulfilling four or more of the American College of Rheumatology (ACR) classification criteria for SLE [20], were recruited from an ongoing prospective control study program at the Department of Rheumatology, Skåne University Hospital, Lund, Sweden. Serum samples were collected from each patient, at a time-point of active disease. Serum samples were centrifuged, frozen, and stored at −80°C within 2 h of venipuncture.

The demographics including treatment with immunosuppressive drugs at the time-point of blood sampling are described in Table 1, where also data on complement and CRP levels, as well as relevant autoantibodies are shown. The SLE patients were classified into three different phenotype groups according to disease severity as previously described [5]: 1) Mild SLE with skin and musculoskeletal involvement (SLE-G1, n = 10); 2) More severe SLE with serositis, systemic vasculitis and/or involvement of central nervous system (CNS) but not kidney involvement (SLE-G2, n = 10); and 3) presence of SLE glomerulonephritis (SLE-G3, n = 10). SLE disease activity was evaluated using SLEDAI-2K [21].

**Table 1:** Demographic data of the SLE patients included in the study at the time-point of blood sampling. SLE-G1: Mild SLE with skin and musculoskeletal involvement, SLE-G2: More severe SLE with serositis, vasculitis, CNS but not kidney involvement, SLE-G3: SLE glomerulonephritis.

**Parameter (N)**	**SLE-G1 (10)**	**SLE-G2 (10)**	**SLE-G3 (10)**
Gender (female:male)	10:0	8:2	8:2
Age at onset ^a^^)^	45 (26–51)	38 (27–46)	28 (24–34)
Disease duration (months) a)	102 (42–179)	79 (15–206)	74 (10–139)
SLEDAI-2K ^b^^,^^c^^)^	6 (4–10)	7 (2–32)	16 (10–32)
Positive ANA ^d^^)^	10	10	10
Anti-DNA (% positive)	10	60	70
Complement protein C1q (%) ^b^^)^	105 (58–217)	109 (0–133)	58 (35–158)
Complement protein C3 (mg/mL) ^b^^)^	1.10 (0.35–1.48)	0.85 (0.37–1.56)	0.59 (0.34–1.22)
Complement protein C4 (mg/mL) ^b^^)^	0.16 (0.09–0.43)	0.13 (0–0.28)	0.11 (0.04–0.33)
CRP (mg/L) ^b^^,^^e^^)^	12.0 (0–89)	13.5 (0–75)	11.5 (0–33)
Treatment			
*Prednisolon*	4	8	10
*Hydroxychloroquine*	5	4	2
*Azathropine*	2	4	1
*Cyclosporine A*	1	0	3
*Cyclophosphamide*	1	0	3
*Methotrexate*	0	0	1
*Intravenous Ig*	0	0	1

a) Median (25th and 75th percentile)

b) Median (range)

c) SLEDAI-2K—Systemic Lupus Erythematosus Disease Activity Index 2000

d) ANA—Antinuclear Antibodies

e) CRP—C-reactive protein

Serum samples from 19 patients with SSc were obtained at their first enrolment for inquiry of the SSc diagnosis at the Department of Rheumatology, Skåne University Hospital, Lund, Sweden, and the demographics are described in Table 2. The SSc patients fulfilled the inclusion criteria of a) a definitive diagnosis of SSc according to the ACR criteria [22]; b) a disease duration of less than five years from the onset of skin involvement; and c) had not been previously treated with any of the following immunosuppressive drugs: azathioprine, chlorambucil, colchicine, cyclophosphamide, cyclosporine A, D-penicillamine, methotrexate or mycophenolate mofetil. The SSc patients were classified according to whether they had limited cutaneous SSc (lcSSc, n = 9) or diffuse cutaneous SSc (dcSSc, n = 10) [23], and the clinical data presented in Table 2 was obtained as previously described [24].

**Table 2:** Demographic data of the SSc patients included in the study.

**Parameter (N)**	**lcSSc (9)**	**dcSSc (10)**
Gender (female:male)	9:0	7:3
Age at onset ^a^^)^	55 (44–70)	47 (34–55)
Disease duration (months) ^a^^)^	32 (27–43)	17 (10–28)
Skin score ^a^^)^	10 (9–12)	17 (10–28)
CRP (mg/L) ^b^^,^^c^^)^	0 (0–140)	6 (0–66)
Organ involvement		
*Esophagus*	9	9
*Lung*	4	7
*Heart*	1	4
*PAH* ^*d*^^*)*^	1	0
*Kidney*	1	1
*Muscle*	0	1
*Joint*	0	1
No. ANA positive ^e^^)^	9	10
No. anti-Scl-70 positive	0	4
No. anti-centromere positive	8	0

a) Median (25th and 75th percentile)

b) Median (range)

c) CRP—C-reactive protein

d) PAH—Pulmonary arterial hypertension

e) ANA—Antinuclear Antibodies

Serum samples from 20 patients with pSS, all fulfilling the American–European consensus criteria [25], were obtained from Department of Rheumatology, Skåne University Hospital, Malmö, Sweden. The demographics are described in Table 3 with data on complement and CRP levels, as well as relevant autoantibodies. None of the pSS patients were treated with any immunosuppressive drugs at the time of blood sampling except for three patients; one was treated with cyclosporine A and low dose prednisone and the other two with only low dose prednisone. All sample aliquots were stored at -80°C.

**Table 3:** Demographic data of the patients with primary Sjögrens syndrome (pSS) included in the study.

**Parameter (N)**	**pSS (20)**
Gender (female:male)	19:1
Age at onset ^a^^)^	61 (45–65)
Disease duration (months) ^a^^)^	132 (120–156)
Complement protein C1q (%) ^b^^)^	123 (30–173)
Complement protein C3 (mg/mL) ^b^^)^	1.3 (0.4–1.6)
Complement protein C4 (mg/mL) ^b^^)^	0.3 (0.02–0.4)
CRP (mg/L) ^b^^,^^c^^)^	5 (0–37)
No. ANA positive ^d^^)^	18
No. anti-SSA positive ^e^^)^	15
No. anti-SSB positive ^f^^)^	12
No. RF positive ^g^^)^	13

a) Median (25th and 75th percentile)

b) Median (range)

c) CRP—C-reactive protein

d) ANA—Antinuclear Antibodies

e) SSA–Sjögren’s-syndrome-related antigen A

f) SSB–Sjögren’s-syndrome-related antigen B

g) RF–rheumatoid factor

For all studied groups of patients normal reference interval were: CRP: <3.0mg/L, C3: 0.77–1.38 g/L, C4: 0.12–0.33 g/L and C1q: 78–131%.

Samples from eighteen healthy female volunteers were also included in the study (mean age: 35 (range 20–53).

### Ethic statement

The study was approved by Lund University regional ethics board (LU-2002/378) and an informed written consent was obtained from all participants.

### Metabolic analysis

#### Sample preparation and analysis

The frozen serum samples from the patients were analyzed according to the protocol of Jiye et al. [26] and then analyzed by gas chromatography coupled mass spectroscopy (GC-MS). An aliquot of 100 μL of thawed serum was added to Sarstedt safety cap tubes and 900 μL of the extraction mixture consisting of methanol:water (9:1) containing 11 stable isotopically labeled internal standards were added. The mixture was shaken for 2 min at 30 Hz and stored in an ice-bath for 2 hours before centrifugation in an Eppendorf centrifuge (Model 5417C) for 10 min at 4°C and14000 rpm. Thereafter, 200 μL of the supernatant was transferred to GC-MS-vials and evaporated to dryness, using a Speedvac. The samples were then derivatized by shaking them for 10 min with 30 μL pyridine containing methoxyamine, 15 μg/μL, followed by incubation at 70°C for 60 min. Following incubation at RT for 16 hours N-Methyl-N-trifluoroacetamide (MSTFA), 30 μL, containing 1% Trimethylchlorosilane (TMCS) was added. The mixture was vortex-mixed and allowed to react for 1 hour before addition of 30 μL of heptane containing 15 ng/μL methyl stearate. The extracted and derivatized samples were placed in a Agilent 7683 Series auto sampler (Agilant, Atlanta, GA) and 1 μL was injected splitless into a Agilent 6980 GC equipped with a 10 m x 0.18 mm i.d. fused-silica capillary column chemically bonded with 0.18 Tm DB5-MS stationary phase (J&W Scientific, Folsom, CA) coupled to a Pegasus III TOFMS (Leco Corp., St Joseph, MI) mass spectrometer. The injector temperature was 270°C, the septum purge flow rate was 20 ml min-1 and the purge was turned on after 60 s. The gas flow rate through the column was 1 ml min-1, the column temperature was held at 70°C for 2 minutes, then increased by 40°C min-1 to 320°C, and held there for 2 min. Ions were generated by a 70 eV electron beam at an ionization current of 2.0 mA, and 30 mass spectra s-1 were recorded in the mass range from m/z 50 to 800, after a solvent delay of 170 s. The ion source was maintained at 200°C. An alkane series (C10-C40) was run together with all samples to enable calculation of retention indices of the metabolites.

#### Data processing

All non-processed MS-files from the metabolic analysis were exported from the ChromaTOF software in NetCDF format to MATLAB™ software 2006b (Mathworks, Natick, MA, USA), in which all data pre-treatment procedures, such as base-line correction chromatogram alignment, data compression and Hierarchical Multivariate Curve Resolution (H-MCR) were performed using scripts as described by Jonsson et al [27,28]. All manual integrations were performed using ChromaTOF 2.12 software (Leco Corp., St Joseph, MI, USA) or in-house MATLAB scripts. The data processing protocols resulted in peak areas for the derivatized metabolites and corresponding mass spectra. The data processing of the GC-MS data using the H-MCR script resulted in a dataset with putative metabolites. All variables, i.e. putative metabolites, were checked manually and variables originating from internal standards and processing artefacts excluded. Additionally, chromatographic peaks originating from one compound but split during the data processing were re-processed using the H-MCR program or by manual integration yielding a dataset with relative levels of 243 putative metabolites.

#### Metabolite identification

Totally 73 of the 243 metabolites could be uniquely identified by comparison of retention indices and mass spectra with data in commercial, as well as in-house, retention indexes and mass spectra libraries using NIST MS Search 2.0 (National Institute of Standards and Technology, 2001). Match values ranking the spectra were calculated using the dot product of the resolved spectrum and the database spectrum weighted with higher m/z peaks having more weight than lower m/z peaks, since higher m/z values are considered to be more compound specific. Match values range from 0–999, with 999 being an identical match. Verification of identification was obtained by combining match values with retention time index (+/- 10 acceptance), calculated based on the analytically characterized alkane series.

#### Data normalization

The dataset of putative metabolites was then normalized with the aid of the 11 added internal standards; a non-centered principal component analysis (PCA) model was built on the basis of the intensity of the internal standards. The magnitude of the PCA model t_1_-score of a given sample was taken as a general measure of the intensity. Differences in intensity between samples are primarily believed to originate from differences in the system inlet efficiency and ion suppression effects [29].

### Statistical methods

All multivariate modelling was performed using SIMCA version 13 (Umetrics, Umeå, Sweden). Principal component analysis (PCA) [30,31] was used for metabolomics data overview using score plots, and SIMCA classification [32] visualized with Cooman’s plots [33] were used to compare subject classification with different PCA models. Furthermore, OPLS-DA [34,35] was used to elucidate the metabolomics differences between various groups of subjects, using the OPLS-DA loadings (p(corr)) to estimate the discrepancies for each metabolite for the compared groups [36]. The OPLS-DA models statistics are reported in Table A in S1 File. Whereas R2X and Q2 values for PCA are not demonstrating class separation ability, but an overview of the main variation (R2X) and indication of the robustness of the model itself (Q2), the OPLS-DA model statistics is targeted on the class-discrimination statistics.

#### SIMCA Classification and Coomans’ plot

The PCA gives residuals, i.e. deviations between the data and the model, named DModX. When these residuals are large, this indicates a deviating behavior. These distances are used for classification in the SIMCA (Soft Independent Modeling of Class Analogy) method [32] by the introduction of a critical distance which determines how far away data can be from the model and still be classified as part of that model (i.e. class). The critical limit for the distance to model measure (DCrit) is determined using an F-test at significance level 0.05. Coomans’ plot [33] was used in presented study to visually illustrate the classification. For each of the class model, the “Leave one out” (LOO) cross validation technique was used [37].

#### Orthogonal projections to latent structures (OPLS) and classification (OPLS-DA)

OPLS [34] is an extension to the supervised PLS [38,39] regression method. OPLS can, analogously to PLS-DA, be used for discrimination (OPLS-DA) [35]. In presented study, threshold for class belonging was set to zero, as described in Kuen *et al*.[40].

#### Significance testing

Model significance was found by means of cross-validation [41]. The sensitivity and specificity for the OPLS-DA model predictions were based on cross-validation estimates and calculated as: Sensitivity = true positive / (true positive+false negative); Specificity = true negative / (false positive+true negative). Significance statistics based on CV-ANOVA [42] for all studied OPLS-DA models are given in Table B in S1 File. Receiver operating characteristic (ROC) curves were calculated in GraphPad Prism 6 (San Diego, CA, USA). For OPLS-DA models they were calculated using predicted cross-validated values for the fitted Y values for observations in the dataset computed with the cross-validation procedure; for the PCA models score values from the first two PCA score vectors were used. P-values for metabolite differences between studied groups of samples were calculated in Excel by performing two-sample unequal variance t-test with two-tailed distribution. Significant p-values (p < 0.05) for between group separations are given in Table C in S1 File.

## Results

### Metabolic profiles of the three SLE phenotypes

The first step in the multivariate analysis of the metabolic data was to investigate if the three different SLE phenotypes G1 (mild SLE), G2 (moderately severe SLE) and G3 (glomerulonephritis, severe SLE) may be differentiated by their metabolic profiles.

**Fig 1:**
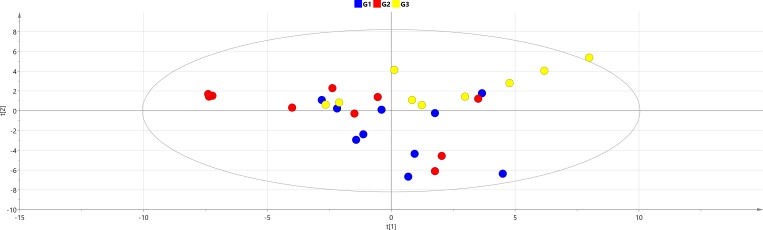
PCA model overview of the three different clinical SLE phenotypes. PCA model parameters: R2X(cum) = 0.522, Q2(cum) = 0.138, 4 components.

An overview PCA model was calculated and the resulting score plot is shown in Fig 1. It is obvious that the metabolic profiles of the three phenotypes overlap. The most severe form of SLE, G3 (glomerulonephritis), showed some difference but even this class overlapped with the other two. Based on the observation that the measured metabolic profiles did not differ to a large extent between the three clinical phenotypes, the whole group of SLE subjects were pooled as one group in the models presented in the following sections.

### Metabolic profiles of controls, SLE and similar diseases

**Fig 2:**
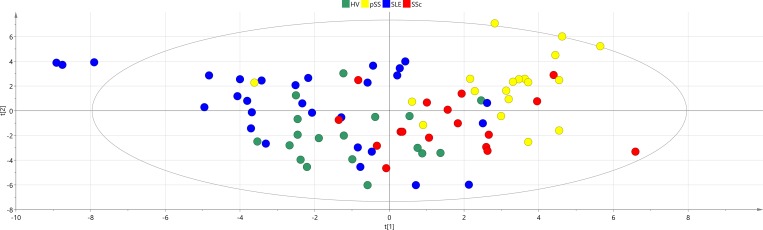
PCA model overview SLE and control group (HV), and the two related disease groups (pSS, SSc). PCA model parameters: R2X(cum) = 0.602, Q2(cum) = 0.158, 8 components.

The second step in the multivariate analysis was to explore characteristics of the metabolic profile of SLE in comparison with two other similar autoimmune diseases: Systemic Sclerosis (SSc) and primary Sjögren’s Syndrome (pSS) and a control group of healthy volunteers (HV). The PCA model score plot (Fig 2) showed that the SLE group could be separated from pSS, SSc and control group (HV), with majority of SLE samples clustering in the upper left quadrant of the PCA plot, although some SLE samples overlapped with other groups as well. To confirm separation between studied groups of samples visible at the PCA plot, ROC curves based on the score values from the first two PCA scores (t_1_ and t_2_) for SLE vs HV, HV vs SSc and SSc vs Pss (that is going from one group to another along the first score vector) and SLE versus other diseases were calculated with following AUC values: SLE vs HV(t_1_) = 0.6264, SLE vs HV(t_2_) = 0.7586, HV vs SSc(t_1_) = 0.8562, HV vs SSc(t_2_) = 0.6307, SSc versus pSS (t_1_) = 0.7430, SSc versus pSS (t_2_) = 0.8266, SLE vs SSc(t_1_) = 0.8702, SLE vs SSc(t_2_) = 0.6836, SLE vs pSS(t_1_) = 0.9456, SLE vs pSS(t_2_) = 0.6098. ROC curve analysis confirmed good (AUC 0.7–0.8) or very good (AUC: 0.8–0.9) discrimination between studied group of samples in at least one direction of the PCA score plot.

**Fig 3:**
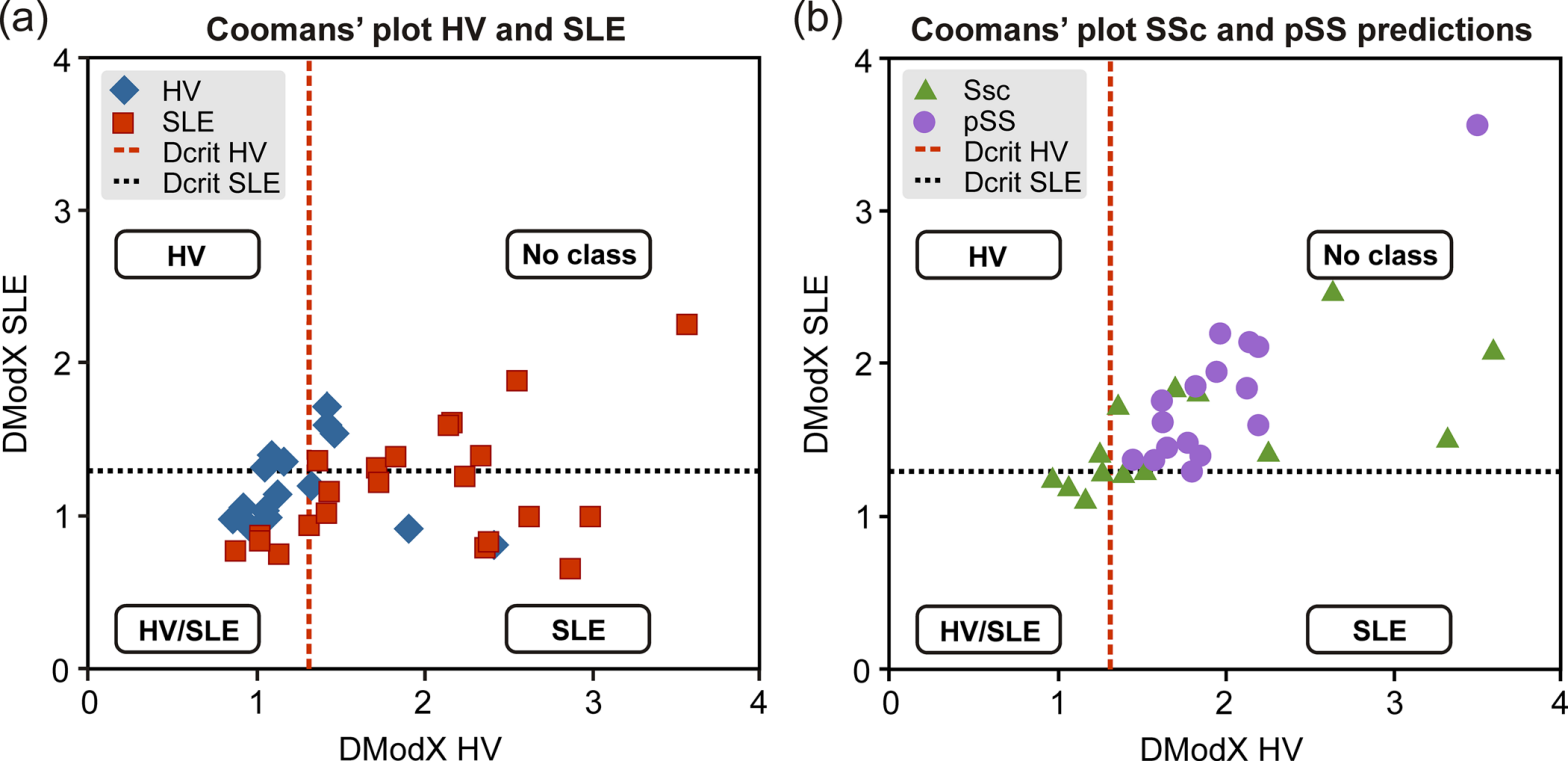
Cooman's plot was used to assess specificity and sensitivity of metabolic profiles to distinguish between A) SLE and control group (HV). B) The same model was used to predict SSc and pSS groups.

Presented results indicated that the metabolic profile of SLE can be distinguished from those of pSS, SSc and controls. Classification of SLE was performed using the SIMCA method. The cross-validation results are illustrated in the Cooman’s plots, see Fig 3. The DModX values revealed the dissimilarity (distance) to the models. In Fig 3A, the SLE and control group (HV) classifications are shown with a sensitivity of 59% for the SLE model and 61% for the control group model. The specificity for the two models was acceptable, 67% for the SLE model and 86% for the HV model. In Fig 3B the results were corroborated as the other disease groups (pSS, SSc) were classified with a specificity of 70% to indicate that there are clear metabolic differences in these otherwise clinically similar rheumatic diseases.

In addition, we tested the performance of the OPLS-DA models by creating receiver operating characteristic (ROC) curves based on predictions obtained from the cross-validation procedure (Fig A in S1 File). The accuracy of each model was estimated as the area under the curve (AUC), which was above 0.95 for all studied models.

**Fig 4:**
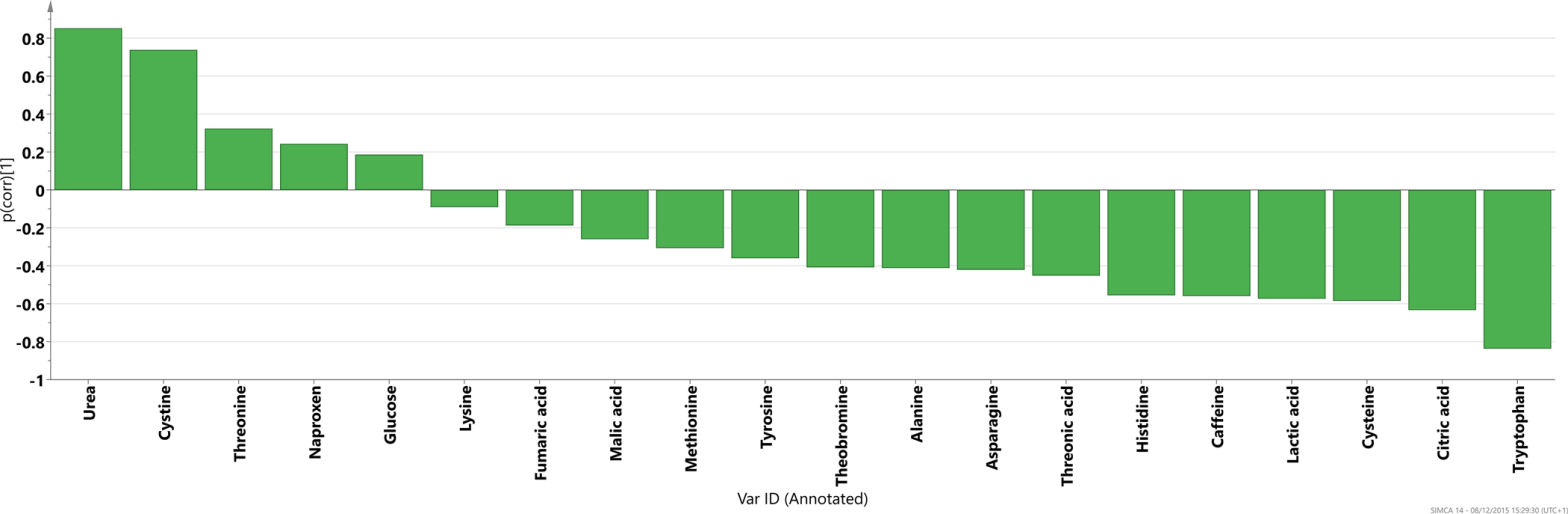
P(corr) profile for the metabolites significant for the separation (p < 0.05) between SLE and control group (HV).

**Fig 5:**
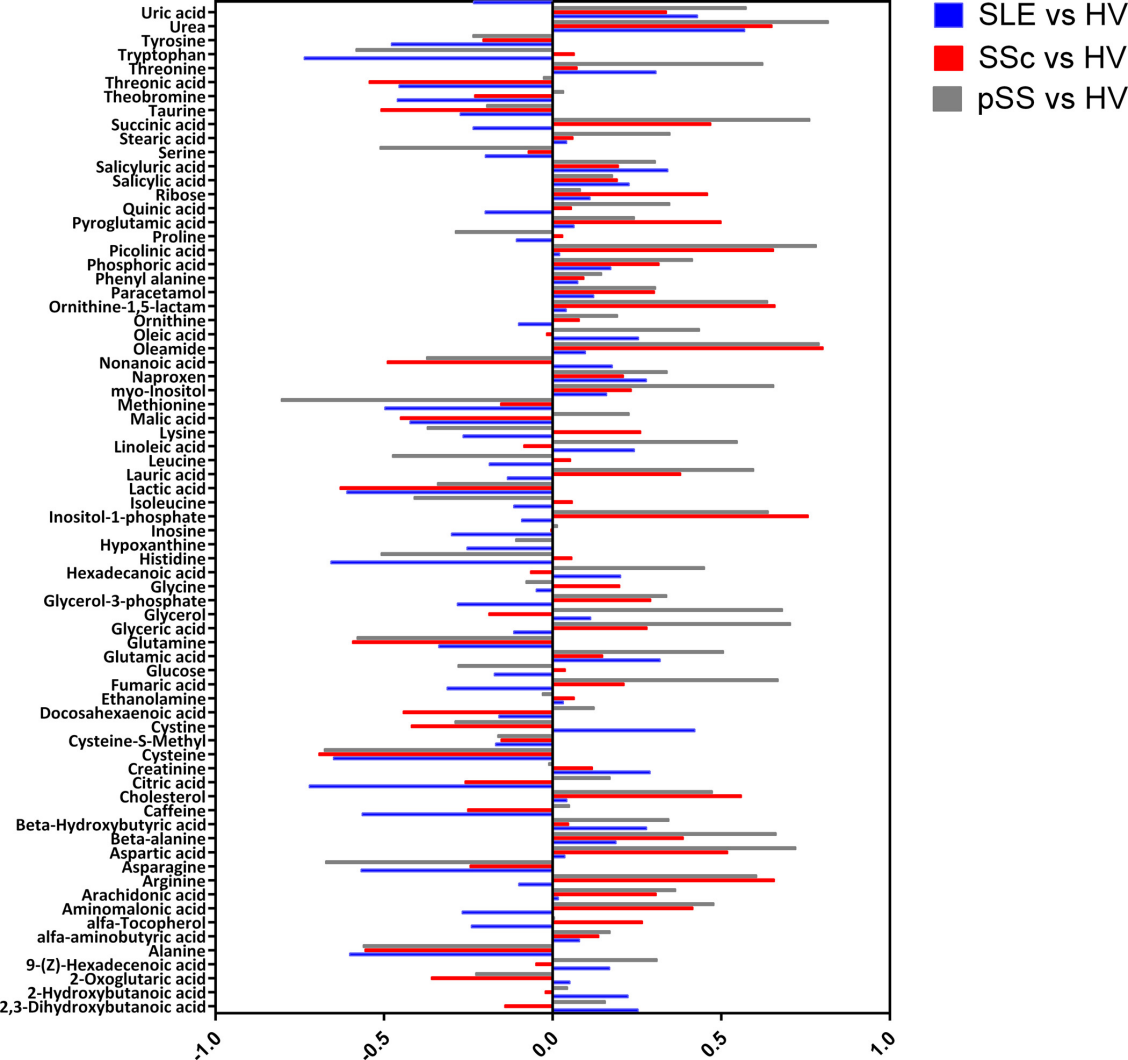
Metabolic profiles (p(corr) values) from OPLS-DA models between SLE versus healthy volunteers (HV) (blue),SSc versus HV (red) and pSS versus HV (gray).

An OPLS-DA model was used to identify which metabolites characterize SLE vs the control group (HV). All SLE samples regardless of phenotype (G1, G2 or G3) were included to represent the full spectrum of SLE. The p(corr) profile of the significant metabolites (p<0.05) is displayed in Fig 4 where levels represent SLE compared to control group. OPLS-DA models were also used to understand the different metabolic profiles between pSS and SSc in relation to the control group (HV) and in comparison to SLE versus HV metabolic profile. In Fig 5 the OPLS-DA metabolic profiles are compared for all three diagnoses that highlight differences versus the control group.

Obtained metabolic profile between SLE patients and healthy volunteers can be used to investigate major pathogenic pathways in SLE.

## Discussion

In SLE, an autoimmune disease with diverse clinical manifestations sharing similarities with related rheumatic diseases, there is a great need to develop better tools for diagnosis as well as to monitor disease activity. Furthermore, the pathogenesis of SLE is not completely understood, partly due to the heterogeneity of the disease and novel approaches to identify underlying pathways contributing to disease are warranted. In this study we used metabolomics to identify metabolites differentially regulated in SLE patients in comparison to healthy volunteers and patients with related rheumatic disorders to provide tools for SLE diagnosis. Based on obtained data we also discuss which underlying biochemical pathways can be activated in SLE.

Metabolomics may have the potential to be used as a measure of disease severity in SLE, considering the ability to partly distinguish the less severe SLE patients (SLE-G1 and SLE-G2) from the more severe lupus nephritis cohort (SLE-G3). The fact that the subgroups could not be completely separated fits well with the clinical situation where e.g. symptoms seen in G1, such as skin and joint manifestations, sometimes are present also in the G3 group with glomerulonephritis. Spread within SLE sub-groups and their overlap could be also influenced by the fact that sub-groups were not perfectly matched based on personal and disease-related parameters, which will always be represented in metabolic data. Because of the not complete separation between SLE subgroups, we decided to merge them into one group in the subsequent analyses.

We next investigated the potential of metabolic profiling as a diagnostic tool. Utilizing a set of 73 uniquely identified metabolites we found a markedly different metabolic profile in SLE patients as compared to healthy individuals (Fig 5A and 5B) with five metabolites significantly up-regulated and fifteen down-regulated in SLE compared to controls according to p-values (Fig 4). These results of the ROC curve analysis of cross-validated predictive scores (Fig A in S1 File) proved excellent accuracy of the applied OPLS-DA modeling of metabolic profiles for the discrimination between studied diseases and controls. Furthermore, the individual models of SLE and healthy individuals showed acceptable classification strength with regard to other diagnoses, with specificity above 67% for all analyses (SLE, pSS and SSc). Selected metabolites were also significantly different between SLE and other diseases, as showed by p-values (Table C in S1 File). It is especially noteworthy that metabolic profiles in comparison to healthy volunteers were different for primary Sjögrens syndrome (pSS), systemic sclerosis (SSc) and SLE, which are rheumatic diseases with overlapping clinical pictures.

We also observed that the SLE pattern of lowered levels of amino acids (nineteen out of twenty six) was not as pronounced in the SSc patients (eleven out of twenty six) which means that the amino groups released from the urea cycle could have been reused to create new amino acids. This can be exemplified by the noted increase in arginine and simultaneous decrease in 2-oxoglutaric acid for SSc patients compared to control group and compared to SLE-HV profile. When comparing the metabolic profile for SLE with the one for pSS it was found that the latter showed an increase in many organic acids compared to controls (in contrast to SLE patients) which could indicate that pSS patients were experiencing a higher degree of oxidative stress. The most visible example of this was fumaric acid which was found significantly lower in the SLE vs control model and significantly higher in the pSS vs control model. Oxidative stress is well described in SLE but we have not been able to find evidence of more pronounced oxidative stress in pSS compared to SLE in the literature. However, this lack of evidence in the literature most likely reflects that this subject is not thoroughly investigated. Observed differences point to different biochemical pathways activated in each disease compared to controls and indicate that metabolomics may be one of the tools which could in the future provide a set of biomarkers useful for diagnosis of SLE.

Since our study was focused on investigation of SLE, we put observed metabolic profile differentiating SLE from controls in a biochemical context. Previous metabolomics study on SLE [43] showed reduced concentrations of several metabolites, including many amino acids in SLE patients compared to controls. In the publication by Wu *et al*. [44] metabolic profiling of sera samples from SLE patients was performed to discriminate between rheumatoid arthritis patients and controls. Their main findings suggest evidence of oxidative stress and reduced energy generation in SLE affecting the amino acid and fatty acid metabolism. Our results confirm this observation, with the majority of amino acids, as well as organic acids down-regulated in SLE patients compared to controls, whereas most of the fatty acids were up-regulated. J. Saegusa et al. [45] reported that 25 metabolites detected in sera from SLE patients were significantly different compared to controls, including glutamic acid and urea, found significant also in our study. Two recent reviews [46,47] summarize current results of ‘omics’ analyses in the SLE field, with only few papers cited in the metabolomics section, clearly demonstrating that there is a need for further exploration of the usefulness of metabolomics in SLE research.

Lower levels of tyrosine and especially tryptophan in SLE patients compared to controls could suggest increased activity of aromatic amino acid decarboxylase (AADC), which transforms these compounds into the trace amine neurotransmitters. Changes of levels of these amino acids in blood could result in altered levels of dopamine and serotonin in the brain which may have effects on the mood and be related to depression symptoms. In fact, a wide range of manifestations from the central nervous system (CNS) can be seen in patients with SLE with unclear pathogenesis [48] and our present results might indicate the involvement of pathways worth further exploration. Lower levels of tryptophan could be also connected to kynurenine pathway, which has been related to activated immune response and involved in many diseases and disorders, including SLE [49,50]. Importantly, we recently described increased activity of the interferon-sensitive enzyme indolemaine 2,3-dioxygenase in SLE patients, skewing the tryptophan metabolism in favor of kynurenine instead of serotonin leading to decreased levels of serotonin, a finding related to disease severity [51]. Interestingly, in MRL/lpr mice, a lupus mouse model, increased depression-like behaviour and visuospatial memory impairment was dependent of activation of the same pathway with corresponding brain levels of serotonin and kynurenine pathway metabolites [52].

The metabolic profile for SLE patients compared to the matched control group showed an increased activity in the urea cycle manifested by decreased levels of arginine and ornithine and increased levels of urea. This could have been a result of breakdown of proteins during catabolic process. Decrease of arginine in SLE patients could also point to an increase in nitric oxide (NO) levels and hence could be connected to increased oxidative stress. We also found an increase of xanthine oxidase activity manifested by decreased hypoxanthine levels and increased level of its oxidation product uric acid. Further, in SLE patients, cystine was increased while cysteine was decreased compared to healthy individuals. The cystine/cysteine ratio can be related to the redox potential of the cell and hence to oxidative stress. Together, these results support increased oxidative stress in SLE patients observed by othersboth in patients with SLE and in lupus-prone mice as summarized in the review by Perl et al [6].

Interestingly, caffeine, theobromine and quinic acid, part of the caffeine metabolism, were all found to be down-regulated in SLE. This could suggest that the SLE patients either consumed less coffee/tea/cocoa or that they metabolized these compounds more efficiently, both statements needing verification. The latter explanation may be due to that paracetamol has been shown to increase metabolism of caffeine [53] as well as the amount of sulphur amino acids [54] resulting in an increased caffeine metabolism and low concentrations of cysteine and methionine as also observed in this study.

A drawback of this study is the use of immunomodulatory treatment and analgetic drugs in the SLE patients, changing the metabolic profile by introducing new metabolites, including naproxen and paracetamol, as well as disrupting immunopathogenic important pathways. Mentioned drugs are less used in pSS and SSc and not at all in the healthy controls. Also, demographic data of the patients (especially age) were different for different diseases studied and hence could also influence the results. Different ages at disease onset and different treatments thus reflect the real life situation. From ethical reasons we could obviously not have stopped treatment of the patients and unfortunately in this study we were not able to perform patient selection which would allow us to match patients based on their personal parameters. Some of the results presented by us may thus be influenced by this differences in age and treatment, but we believe that our main conclusions are not changed.

## Conclusions

The present investigation shows that metabolomics has the potential to be used as a diagnostic tool for differentiating SLE from healthy controls as well as from other similar immunological diseases such as SSc and pSS. In particular, our data clearly demonstrate prevalent oxidative stress as well as abnormal tryptophan metabolism through AADC or the kynurenine pathway in SLE patients. If validated in future studies, our observation may lead to much needed novel diagnostic and therapeutic options in SLE patients.

## Supporting Information

S1 File Supplementary information. (DOC)
