# Supplementary material for: Metabolic Profiling of Systemic Lupus Erythematosus and Comparison with Primary Sjögren’s Syndrome and Systemic Sclerosis
Source: PLoS One. 2016 Jul 21;11(7):e0159384. doi: 10.1371/journal.pone.0159384 (PMC4956266; doi:10.1371/journal.pone.0159384)
Supplement: S1 File — (DOC) [file pone.0159384.s001.doc]

# Supplementary information

# Metabolic profiling of systemic lupus erythematosus (SLE) and comparison with primary Sjögren´s syndrome (pSS) and systemic sclerosis (SSc)

## Anders A Bengtsson, Johan Trygg, Dirk Wuttge, Gunnar Sturfelt, Elke Theander, Magdalena Donten, Thomas Moritz, Christian Lood, Carl-Johan Sennbro, Frida Torell, Stefan Rännar, Torbjörn Lundstedt

## Model Statistics

All OPLS-DA models were very good in separating the classes of objects. In table S1 the OPLS-DA model statistics are reported and in Table S2 some additional statistical measurements are listed.

- **Observations:** Samples in first class **+** Samples in second class (see Model name for classes)
- **Components:** (Predictive components **+** y-orthogonal components **+** x-orthogonal components)
- **R2X:** Predictive variance in X matrix
- **R2Xyo:** y-orthogonal variance in X matrix
- **R2Y:** Explained variance of Y-vector
- **Q2Y:** Predicted variance of Y-vector (from cross validation)
- **p-value:** Probability value from t-test of the calculated Y-values. (YPred of training set)
- **Sensitivity:** True prediction rate (TPR), level of correctly classified positive samples. Diagnosed disease samples or active SLE are used as positive samples.
- **Specificity:** Level of correctly classified negative samples. Healthy or inactive SLE are used as negative samples.

**Table A.** Model statistics for all OPLS-DA models used in the investigation.

| **No.** | **Model name** | **Observations** | **Components** | **R2X** | **R2Xyo** | **R2Y** | **Q2Y** |
| --- | --- | --- | --- | --- | --- | --- | --- |
| M1 | HV vs All SLE | 18+29 | (1+1+0) | 0,0998 | 0,14 | 0,712 | 0,516 |
| M2 | HV vs pSS | 18+19 | (1+0+0) | 0,205 | - | 0,764 | 0,703 |
| M3 | HV vs SSc | 18+17 | (1+1+0) | 0,126 | 0,0827 | 0,92 | 0,78 |
| M4 | HV vs SLE-G1 | 18+10 | (1+0+0) | 0,139 | - | 0,614 | 0,374 |
| M5 | HV vs SLE-G2 | 18+10 | (1+1+0) | 0,155 | 0,157 | 0,83 | 0,561 |
| M6 | HV vs SLE-G3 | 18+9 | (1+1+0) | 0,122 | 0,0875 | 0,962 | 0,757 |

***Table B.*** *Model significance for all OPLS-DA models used in the investigation.*

| **No.** | **Model name** | **p-value** | **Sensitivity** | **Specificity** |
| --- | --- | --- | --- | --- |
| M1 | HV vs All SLE | <0.001 | 97% | 100% |
| M2 | HV vs pSS | <0.001 | 95% | 100% |
| M3 | HV vs SSc | <0.001 | 100% | 100% |
| M4 | HV vs SLE-G1 | <0.001 | 90% | 94% |
| M5 | HV vs SLE-G2 | <0.001 | 100% | 100% |
| M6 | HV vs SLE-G3 | <0.001 | 100% | 100% |

**Table C.** Significant p-values (below 0.05) for the between group comparison of metabolic levels.

|  | **SLE versus HV** | **SLE versus SSc** | **SLE versus pSS** | **SSc versus HV** | **pSS versus HV** |
| --- | --- | --- | --- | --- | --- |
| 2,3-Dihydroxybutanoic acid | NS | NS | NS | NS | NS |
| 2-Hydroxybutanoic acid | NS | NS | NS | NS | NS |
| 2-Oxoglutaric acid | NS | NS | NS | NS | NS |
| 9-(Z)-Hexadecenoic acid | NS | NS | NS | NS | NS |
| Alanine | < 0.001 | NS | NS | 0.009 | 0.016 |
| alfa-aminobutyric acid | NS | NS | NS | NS | NS |
| alfa-Tocopherol | NS | 0.007 | NS | NS | NS |
| Aminomalonic acid | NS | 0.005 | < 0.001 | 0.018 | 0.006 |
| Arachidonic acid | NS | 0.024 | 0.003 | 0.033 | 0.005 |
| Arginine | NS | < 0.001 | < 0.001 | < 0.001 | < 0.001 |
| Asparagine | < 0.001 | 0.021 | NS | NS | 0.006 |
| Aspartic acid | NS | 0.005 | < 0.001 | < 0.001 | < 0.001 |
| Beta-alanine | NS | NS | NS | 0.017 | < 0.001 |
| Beta-Hydroxybutyric acid | NS | NS | NS | NS | NS |
| Caffeine | 0.009 | NS | 0.004 | NS | NS |
| Cholesterol | NS | < 0.001 | 0.003 | < 0.001 | 0.004 |
| Citric acid | < 0.001 | < 0.001 | < 0.001 | NS | NS |
| Creatinine | NS | NS | NS | NS | NS |
| Cysteine | < 0.001 | NS | NS | < 0.001 | 0.001 |
| Cysteine-S-Methyl | NS | NS | NS | NS | NS |
| Cystine | 0.003 | 0.000 | NS | 0.007 | NS |
| Docosahexaenoic acid | NS | NS | NS | NS | NS |
| Ethanolamine | NS | NS | NS | NS | NS |
| Fumaric acid | 0.045 | < 0.001 | < 0.001 | NS | 0.002 |
| Glucose | 0.046 | NS | NS | NS | NS |
| Glutamic acid | NS | NS | 0.042 | NS | 0.002 |
| Glutamine | NS | 0.001 | 0.029 | < 0.001 | 0.002 |
| Glyceric acid | NS | 0.002 | < 0.001 | NS | 0.001 |
| Glycerol | NS | NS | 0.005 | NS | < 0.001 |
| Glycerol-3-phosphate | NS | 0.004 | < 0.001 | NS | 0.014 |
| Glycine | NS | NS | NS | NS | NS |
| Hexadecanoic acid | NS | NS | NS | NS | 0.048 |
| Histidine | < 0.001 | < 0.001 | 0.004 | NS | NS |
| Hypoxanthine | NS | NS | NS | NS | NS |
| Inosine | NS | NS | NS | NS | NS |
| Inositol-1-phosphate | NS | < 0.001 | < 0.001 | < 0.001 | < 0.001 |
| Isoleucine | NS | 0.019 | NS | NS | NS |
| Lactic acid | 0.002 | NS | NS | 0.002 | 0.013 |
| Lauric acid | NS | 0.002 | 0.000 | 0.016 | < 0.001 |
| Leucine | NS | 0.010 | NS | NS | NS |
| Linoleic acid | NS | NS | 0.008 | NS | 0.005 |
| Lysine | 0.042 | 0.002 | NS | NS | NS |
| Malic acid | 0.015 | NS | 0.026 | 0.017 | NS |
| Methionine | 0.004 | 0.004 | NS | NS | 0.004 |
| myo-Inositol | NS | NS | 0.006 | NS | 0.002 |
| Naproxen | 0.025 | 0.034 | 0.025 | NS | NS |
| Nonanoic acid | NS | 0.003 | 0.011 | 0.008 | NS |
| Oleamide | NS | 0.004 | < 0.001 | < 0.001 | < 0.001 |
| Oleic acid | NS | NS | NS | NS | NS |
| Ornithine | NS | NS | 0.022 | NS | NS |
| Ornithine-1,5-lactam | NS | < 0.001 | < 0.001 | < 0.001 | < 0.001 |
| Paracetamol | NS | NS | NS | NS | NS |
| Phenyl alanine | NS | NS | NS | NS | NS |
| Phosphoric acid | NS | NS | NS | NS | 0.005 |
| Picolinic acid | NS | < 0.001 | < 0.001 | < 0.001 | < 0.001 |
| Proline | NS | NS | NS | NS | NS |
| Pyroglutamic acid | NS | < 0.001 | 0.017 | 0.001 | 0.040 |
| Quinic acid | NS | NS | 0.004 | NS | 0.047 |
| Ribose | NS | 0.018 | NS | 0.010 | NS |
| Salicylic acid | NS | NS | NS | NS | NS |
| Salicyluric acid | NS | NS | NS | NS | NS |
| Serine | NS | NS | NS | NS | NS |
| Stearic acid | NS | NS | NS | NS | NS |
| Succinic acid | NS | < 0.001 | < 0.001 | 0.004 | < 0.001 |
| Taurine | NS | NS | NS | 0.021 | NS |
| Theobromine | 0.034 | NS | 0.003 | NS | NS |
| Threonic acid | 0.013 | NS | 0.013 | 0.001 | NS |
| Threonine | 0.045 | 0.032 | NS | NS | 0.021 |
| Tryptophan | < 0.001 | < 0.001 | NS | NS | NS |
| Tyrosine | 0.016 | 0.025 | NS | NS | NS |
| Urea | < 0.001 | NS | 0.024 | < 0.001 | 0.003 |
| Uric acid | NS | NS | 0.036 | 0.027 | 0.002 |
| Valine | NS | NS | NS | NS | NS |

A)

B)

C)

Figure A. ROC curves calculated from the OPLS-DA models from predicted values of the fitted Ys for observations in the dataset, computed from the cross validation procedure: (A) represents SLE versus healthy volunteers (HV) (AUC=0.9521, Std. Error = 0.0279, 95% confidence interval 0.8974 to 1.007, p value < 0.0001), (B) pSS versus HV (AUC=0.9921, Std. Error = 0.0107, 95% confidence interval 0.9702 to 1.012, p value < 0.0001), (C) SSc versus HV (AUC=1.000, Std. Error = 0.0, 95% confidence interval 1.000 to 1.000, p value < 0.0001); false positive rate at x axis and true positive rate at y axis, using class-belonging values predicted by the OPLS-DA models.
